# Assessment of Thyroid Hormones Using the Immulite 2000xpi Analyzer in Healthy Donkeys

**DOI:** 10.3390/vetsci13070690

**Published:** 2026-07-15

**Authors:** Carmen Davias, Alejandro Perez-Ecija, Daniela Ruiz-Lastra, Adelaida De Las Heras, Ramiro Toribio, Francisco J. Mendoza

**Affiliations:** 1 Department of Animal Medicine and Surgery, University of Cordoba, 14014 Cordoba, Spain; carmendaviasm@gmail.com (C.D.); danielaruizlastra19@gmail.com (D.R.-L.); v02hesaa@uco.es (A.D.L.H.); fjmendoza@uco.es (F.J.M.); 2 Department of Veterinary Clinical Sciences, College of Veterinary Medicine, The Ohio State University, Columbus, OH 43210, USA; toribio.1@osu.edu

**Keywords:** chemiluminescent assay, donkeys, endocrinology, horses, immulite

## Abstract

The Immulite 2000xpi, a chemiluminescent immunoassay (CLIA)-based analyzer, is currently recommended for measuring thyroid hormones (TH) concentrations in equids. However, scarce data are available on TH measurements in donkeys using this technique. Similarly, the effects of factors such as age and gender on TH concentrations have not been assessed with this analyzer. Serum free and total T4 and T3 concentrations were measured in 40 healthy donkeys (6.1 ± 0.8 years old; 8 intact jacks and 32 jennies) and 41 horses (10.7 ± 0.5 years old; 15 stallions and 26 geldings) using the Immulite 2000xpi. A Mann–Whitney test was used to compare two groups, and a Kruskal–Wallis test followed by Dunn’s post hoc test was used for comparisons among more than two groups. This analyzer had lower intra-assay variability and fewer measurements under the detection limit for tT4 and fT4 than for tT3 and fT3 determinations. The canine TSH kit for thyroid-stimulating hormone (TSH) showed high imprecision. Donkeys had higher serum TH concentrations than horses. No differences were observed among gender or age groups. This is the first study to report TH concentrations in donkeys using a CLIA-based analyzer and to evaluate the effects of several factors on TH concentrations. The canine TSH kit is not valid for measuring TSH in equids.

## Simple Summary

Novel biochemical analyzers such as the Immulite 2000xpi (Siemens Healthineers, Erlangen, Germany) are recommended by the current equine thyroid hormone (TH) diagnostic guidelines. However, the assessment of TH measurement using this analyzer has not been reported in donkeys. This analyzer had an acceptable consistency for free and total T4 but poor intra-assay precision for determining free and total T3 concentrations, and it does not appear to be valid for measuring thyroid-stimulating hormone (TSH) in this species.

## 1. Introduction

Primary thyroid gland diseases (hypo- or hyperthyroidism) are uncommon in equids [1,2]. Neoplasia and non-neoplastic hyperplastic goiter are the most commonly described thyroid diseases in horses, especially in older animals [3]. The prevalence of benign or malignant enlargement of the thyroid gland appears to be lower in donkeys, despite their longer life expectancy compared to horses [2,4,5]. Congenital hypothyroidism–dysmaturity syndrome has also been reported in donkeys [6]. Similar to horses and foals [7,8], non-thyroidal illness syndrome (NTIS) should be taken into consideration in donkeys [5]. Thus, in any equid, the interpretation of baseline serum thyroid hormone (TH) measurements should be based on species-specific ranges, caution should be taken with isolated values, and diagnosis should be supported by dynamic testing [9]. Clinical signs in adult donkeys suffering from hypothyroidism (the most common thyroid gland disorder) are unspecific and vague, with the exception of goiter, and are similar to those reported in horses [1,9]. Briefly, decreased heart and respiratory rates, decreased body temperature and cold intolerance, coarse coat, poor performance, dullness, and erratic appetite are some of the reported clinical signs.

Serum TH concentrations are affected by multiple factors, including species, gender, age, exercise, physiological status, transport, season, stress, and previous medications [1,10]. In addition, TH concentrations differ among laboratories, techniques, and analyzers; therefore, it is advisable to use ranges generated with each analyzer and avoid extrapolating values between techniques [11]. In this sense, reference ranges in donkeys have previously been published for free triiodothyronine (fT3), total triiodothyronine (tT3), free thyroxine (fT4), and total thyroxine (tT4) using radioimmunoassay (RIA) and enzyme-linked immunosorbent assay (ELISA) [10,12], as well as for reverse triiodothyronine (rT3) using RIA [10] and tT4 using chemiluminescence immunoassays (CLIA) [13].

Due to good laboratory practices, RIA-based techniques are being replaced by other techniques with lower risks and regulations, such as ELISA, CLIA, and electrochemiluminescence assays (eCLIA). Although equilibrium dialysis is considered the gold standard technique for fT4 measurement [14], this method is time-consuming, expensive, and requires specialized handling and analyzers (e.g., liquid chromatography or RIA). Recent guidelines for the diagnosis of thyroid gland diseases in equids recommend the Immulite, a CLIA-based analyzer, as a feasible method for TH measurement [15], and reference ranges are available for horses. At present, only ranges for tT4 have been reported with this analyzer in 16 donkeys of several breeds [13]. Therefore, the aims of this study were: (a) to measure TH concentrations in healthy donkeys using the Immulite 2000xpi analyzer; (b) to compare the results in donkeys with those from a population of healthy horses; and (c) to evaluate the influence of age and gender on TH concentrations using this technique.

## 2. Materials and Methods

### 2.1. Animals

Blood samples were collected from 40 healthy donkeys (6.1 ± 0.8 years old; 8 intact jacks and 32 jennies, 17 of which were pregnant). No animal younger than six months of age was included in this study. All animals were considered healthy based on their clinical history, physical examination (heart and respiratory rates, temperature, mucous membrane color, capillary refill time, intestinal motility, and digital pulses), and a complete hemogram and chemistry profile. Thirty-nine donkeys were of the Andalusian breed, and one was of the Catalonian breed. All animals received humane care in compliance with the Spanish Guide for the Care and Use of Laboratory Animals.

In order to evaluate the correct function and handling of the analyzer and discard any technical effect on the results, a small group of horses was also included in the study to compare the results obtained in our laboratory with those previously published using the Immulite 2000xpi. Forty-one adult male horses (10.7 ± 0.5 years old; 15 stallions and 26 geldings) were included. Horses fulfilled the same requirements explained for donkeys in order to be considered healthy. Regarding horse breeds, twenty-one were Andalusians, nine were Spanish Sport Horses, seven were Hanoverians, and four were Dutch Warmbloods.

### 2.2. Blood Sample Handling and Determinations

Blood samples were collected by jugular venipuncture into serum separator tubes to determine serum TH concentrations and a biochemistry profile panel, or into EDTA-K_3_-containing tubes for complete hematology. Samples were kept cold from collection to laboratory arrival (less than 6 h). Hematology was performed immediately upon arrival using an automatized laser cytometry-based analyzer (Sysmex XN-1000V analyzer, Sysmex Corporation, Kobe, Japan). Serum tubes were immediately centrifuged at 3500 rpm for 5 min at 4 °C, after which the serum was aliquoted into 1.5 mL tubes and frozen at −20 °C until measurements.

The following biochemical parameters were determined using one serum aliquot (Spin 640 Plus, Spinreact, Barcelona, Spain): glucose, triglycerides, urea, creatinine, total proteins, albumin, total bilirubin, gamma-glutamyl transferase, glutamate dehydrogenase, aspartate aminotransferase, creatine kinase, alkaline phosphatase, and lactate dehydrogenase. Globulin concentrations were calculated according to previously reported formulas.

The following THs were measured using another serum aliquot: fT3, tT3, fT4, tT4, and thyroid-stimulating hormone (TSH). Hormone concentrations were determined by CLIA (Immulite 2000xpi, Siemens, Erlangen, Germany), as previously described in horses for tT4 and tT3 [16,17], using dedicated reagents (Siemens Healthcare Diagnostics Products GmbH, Dulsburg, Germany). Assay performance for each kit according to the manufacturer and previous reports [16,17,18] is compiled in Table 1. All TH determinations were performed using the same serum aliquot and under the same thaw cycle, since the Immulite 2000xpi analyzer is available to run multiple determinations simultaneously. If repetition of a measurement was needed, then a new serum aliquot was used.

### 2.3. Statistical Analysis

Normality was assessed by the Shapiro–Wilk test, and results were expressed as mean ± standard error (SE) or median and interquartile range (IQR: 75th–25th percentiles), according to normality. Percentiles were calculated using Tukey’s Hinges method, and outlier values were determined by Tukey’s IQR Fences approach (lower or upper quartile ± 1.5 times the IQR). Outlier results were not excluded since the 5% trimmed mean did not change significantly. Differences between species or the effect of gender were compared using the Mann–Whitney test. When more than two groups were compared (age effect), a Kruskal–Wallis test followed by a Dunn’s post hoc test was carried out. Pearson’s (parametric) or Spearman’s (non-parametric) coefficients were used to determine correlations between parameters. Since it has been demonstrated that age and sex can affect TH concentrations in donkeys and horses [10,19], both variables were adjusted using Quade non-parametric ANCOVA analysis to compare any inferences in TH concentrations.

Samples resulting in concentrations under the limit of detection (LOD) were measured in duplicate using a new aliquot in order to discard any possible handling problem. Those TH measurements under the LOD were not included in the statistical analysis. One sample was measured five consecutive times, and the coefficient of variation was calculated as standard deviation/mean x 100 to determine the intra-assay precision.

Donkeys and horses were grouped into the following three age groups: Group 1: <5 years old, Group 2: 5–10 years old, and Group 3: >10 years old. In addition, the effect of age was also studied as a continuous variable.

Data analyses were performed using dedicated statistical packages (IBM SPSS Statistics 27, IBM Corporation, Armonk, NY, USA; GraphPad Prism 9, San Diego, CA, USA). A *p* < 0.05 was considered significant.

## 3. Results

### 3.1. Animals and Intra-Assay CVs for TH Measurements in Donkeys and Horses Using the Immulite 2000xpi

All donkeys and horses included in this study were healthy according to physical exploration and blood profile results (Tables S1 and S2).

Intra-assay CVs for tT4, fT4, tT3, fT3 and TSH were 4.7%, 3.3%, 5.8%, 5.1% and 28.6% for donkeys, and 6.1%, 7.6%, 9.4%, 8.8% and 37.1% for horses, respectively.

Some TH measurements in both donkeys and horses were under the limit of detection (LOD) with this analyzer. Regarding tT4, one horse (1/41, 2.4%) had a concentration under the LOD (<0.5 µg/dL), and three horses (3/41, 7.3%) had fT4 concentrations under the LOD (<0.3 ng/dL). No donkey had tT4 or fT4 concentrations under the LOD. In contrast, 9/40 (22.5%) donkeys and 13/41 (31.7%) horses, and 14/40 (35.0%) donkeys and 8/41 (19.5%) horses had tT3 (<40 ng/dL) and fT3 (<1 pg/mL) concentrations below the LOD, respectively.

**Figure 1:**
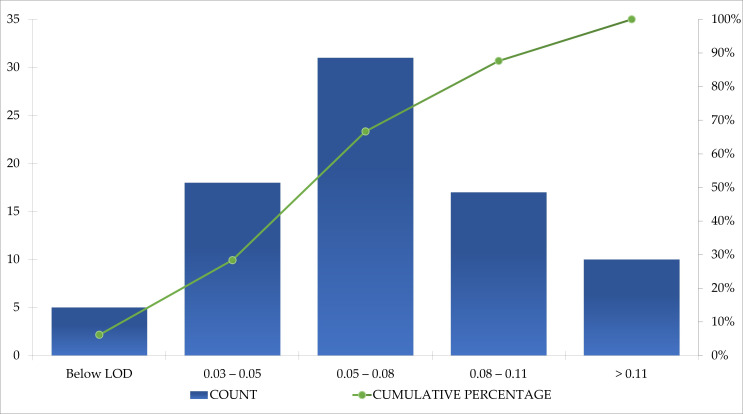
Frequency histogram of TSH results in equids (ng/mL), representing the number of samples with certain results (including those below the LOD) and the cumulative percentage line (green line).

For TSH, 17 out of 40 donkey samples were below the reportable range (<0.03 ng/mL), but 14 gave results when the samples were run in duplicate. For horses, 4/41 samples were under the LOD, but 2/41 yielded a result upon second measurement. However, due to the high imprecision of the TSH assay for both species (CVs > 28%), the results were considered unsuitable for this hormone, and statistical analysis was not performed. A frequency histogram of TSH concentrations in both species is presented in Figure 1.

### 3.2. TH Concentrations in Donkeys and Horses

TH concentrations in donkeys and horses were non-normally distributed and are listed in Table 2. Donkeys had significantly (*p* < 0.05) higher serum tT4, fT4, and tT3 concentrations than horses. No differences were observed for fT3 between both species (Table 2): When comparisons with horses were age- and gender-adjusted, the results of the Quade ANCOVA analysis for both co-variables demonstrated no changes in the significance of tT4 and fT4 (Table 2).

### 3.3. Effect of Age on TH Concentrations in Healthy Donkeys Analyzed by the Immulite 2000xpi

No statistical differences among age groups were detected in TH concentrations in donkeys (Table 3). However, a negative correlation was observed between age and tT4 (r = −0.35, *p* = 0.033) and fT4 (r = −0.31, *p* = 0.05) concentrations. In horses, only a negative correlation was observed between fT4 concentration and age (r = −0.31, *p* = 0.05).

### 3.4. Effect of Gender on TH Concentrations in Healthy Donkeys Analyzed by the Immulite 2000xpi

No significant differences were found between jacks and jennies for any TH (Table 4). Significant correlations between gender and TH were not found. Since only male horses were included in this study, this analysis was not performed in this species.

When animals were classified according to sexual status, non-pregnant jennies had higher (*p* = 0.012) serum tT4 concentrations compared to pregnant ones (Table S3). In horses, no statistical differences were found between stallions and geldings (Table S3). Since only intact donkeys were included, this analysis was not carried out in this species.

## 4. Discussion

Although the CLIA technique has previously been used for the diagnosis of primary hypothyroidism in a case report of a jenny (tT3 and tT4 concentrations) [6], and tT4 values have been reported in 16 donkeys of several breeds to evaluate the effects of the autumn and spring seasons [13], our study evaluates for the first time the suitability of this technique and analyzer for fT4, tT3, fT3, and TSH, as well as the effects of gender and sex on TH concentrations. This analyzer is currently recommended for diagnosing thyroid disorders in the guidelines of the Equine Endocrinology Group [15], and it is commonly used to measure TH in horses in clinical settings [20,21].

TH concentrations in equids can be measured via CLIA, ELISA, or RIA [15]. Nowadays, the use of RIA-based assays is declining due to the requirement of radioisotope-labeled antibodies, specialized training, equipment, and more stringent safety and regulatory measures. ELISAs are available for horses [22] and have been used in donkey blood and milk [23]. Comparatively, CLIA is a less time-consuming and more automated technique than RIA or ELISA and can be safely performed by technicians with less supervision.

While the recommended allowable total error is not available for veterinary endocrinology assays to date, most authors agree that CVs should be lower than 10–20% for a technique to be considered acceptable [24,25,26]. Coefficients of variation for the tT4, fT4, tT3, and fT3 assays were <6% and 10% in donkeys and horses, respectively. Therefore, the use of these assays appears to be suitable in donkeys, similar to previous reports in horses [16,17].

On the other hand, CVs for TSH were >28% in both species. Although we did not perform a complete analytical validation of this assay (which would have required linearity, repeatability, precision within and between runs, recovery, interference studies, comparison with other techniques, etc.), precision is a major component of the total analytic error of a technique [26]. Thus, the canine-specific CLIA-based TSH assay should not be used until a complete validation has been performed in equids.

An assay for equine TSH is not commercially available and, according to some authors, anti-TSH antibodies used to measure TSH in other species do not cross-react with equine TSH [1,15]. We previously investigated two TSH human-specific and canine-specific RIA assays in donkeys, with results being unreliable, similarly to those presented in the present work with a canine CLIA [10]. These findings could be due to a lack of cross-reactivity between the canine/human antibodies and equid samples (donkeys and horses), or to nonspecific binding to other serum compounds (e.g., proteins). It is worth noting that one study used a customized RIA based on anti-TSH antiserum and highly purified equine TSH, which is not commercially available. This assay demonstrated adequate sensitivity and a lack of cross-reactivity with other equine pituitary hormones [27]. There is also a report evaluating variations in equine TSH concentrations using an ELISA, but validation of this kit was not performed [28]. Therefore, due to the high imprecision of the canine-specific TSH assay in the Immulite 2000xpi, this assay should not be used to diagnose primary thyroid gland disorders in equids, since an erroneous diagnosis could be obtained.

In our results, a high number of tT3 and fT3 determinations were under the LOD in both species, mostly in horses. The handling of values falling below an assay’s LOD is a common challenge in many disciplines, including veterinary clinical pathology. Several techniques have been used for dealing with these censored data, such as substitution (with zero, LOD/2, or LOD/√2), multiple imputation, the Tobit regression model, parametric modeling, rank-based non-parametric methods, and Bayesian methods [29,30]. These methods are particularly useful when reference intervals are calculated in order to obtain a complete overview of the population. Since we did not calculate these ranges, and all of the mentioned methods could cause some degree of bias, we decided not to include these data in the statistical analysis and instead only describe the number of determinations below the LOD. A similar approach was used in a previous study using this analyzer to evaluate the effect of levothyroxine sodium on serum tT4 and tT3 concentrations in horses [16].

One study using CLIA in horses reported TH values below the LOD in healthy animals in a similar proportion to our results [16]. Notably, we measured these samples twice using different aliquots in order to discard any technical error from the analyzer. Therefore, in healthy animals, and as previously recommended by other authors [16], low concentrations or results under the LOD with these assays should be interpreted with caution and should not be considered an indicator of hypothyroidism. To confirm hypothyroidism, based on recently reported equine guidelines [15], the fT4 concentration should be determined by equilibrium dialysis and, ideally, a dynamic test (thyrotropin-releasing hormone [TRH] stimulation test) should be performed. Since TRH stimulation would increase tT3 concentrations in healthy animals, this dynamic test could circumvent the limitations of these assays. Dynamic tests (TRH stimulation and tT3 suppression) are recommended for the diagnosis of thyroid disorders in equids [1]; however, to our knowledge, they have not been evaluated in donkeys.

Healthy donkeys had higher serum TH concentrations than healthy horses. Similar findings were observed in a recent study using the Immulite 2000, where donkeys also had higher tT4 concentrations than horses in all seasons [13]. Regarding fT4, tT3, and fT3 measured using the Immulite or other CLIA analyzers, data for donkeys are not available for comparison. When our donkey results are compared with previous reports in healthy horses using the Immulite, tT4 concentrations were also higher [16,17,31], whereas tT3 concentrations were similar [16] or lower [17,31], depending on the report. Our horses were within the reference ranges previously reported for these hormones in this species [16,17,20,31]. No information on fT3 and fT4 measured using CLIA in horses is available for comparison. Several hypotheses have been proposed to explain the dissimilarities in TH concentrations between donkeys and horses, such as differences in thyroid-binding protein concentrations, thyroid-binding protein affinity, hormone half-life, TRH or TSH serum concentrations affecting hypophysis or thyroid gland function, thyroid gland sensitivity to TSH, and peripheral T4 conversion [10]. Donkeys have a higher prevalence of dyslipidemia compared to horses [5]. Whether this finding could be related to higher serum TH concentrations, triggering a rapid and increased fatty acid mobilization from adipose tissue in this species, remains to be elucidated. In this sense, differences in metabolic rate (including differences in body size) between both species could also influence basal serum TH concentrations. Nonetheless, it is important to take into consideration that these differences could also be a species-specific dissimilarity without a functional explanation.

In comparison with other analytical techniques, similar species-related differences were previously reported by our research group using the RIA technique [10]. Serum tT4, tT3, and fT3 concentrations in this study were lower in both donkeys and horses compared to those previously reported by our research group using RIA [10]. In contrast, fT4 concentrations were higher than those obtained with RIA in both donkeys and horses [10]. Reference intervals for TH depend on the technique and laboratory, which prevents any extrapolation and limits comparisons between them [11]. Emphasizing differences among laboratories, similarities and discrepancies have been observed in other studies using RIA in horses [32,33,34,35]. For example, tT4 was higher and tT3 was lower in our donkeys compared to healthy horses [36]. Regarding ELISA, blood TH concentrations in our study were lower compared to those reported in a previous study of donkeys using ELISA [12]. Regarding horses, tT4 and fT4 concentrations measured using ELISA were lower compared to those observed in donkeys in our study, whereas tT3 and fT3 concentrations were higher [19].

Low blood TH concentrations can often be falsely detected in equids, with primary hypothyroidism and NTIS being sporadically diagnosed in donkeys and horses [6,8,9,37]. In addition, multiple factors have been described to decrease TH concentrations in horses, including species, age, gender, season, time of day, sexual status, fasting, exercise, and drug administration (e.g., glucocorticoids and phenylbutazone) [1,10,19,38,39,40,41,42,43]. In contrast, transport and some diets increase TH concentrations in horses [44,45,46]. To the authors’ knowledge, only the effects of species, age, season, breeding season, gender, sexual stage, and transport have been described in donkeys [10,47,48,49]. Similar to previous studies in donkeys using RIA [22], our study did not find any significant sex-related differences. In contrast, previous studies using ELISA have reported that mares have either higher tT4 [50] or all TH concentrations than stallions [19]. Nonetheless, due to the small group of jackasses included in this study, the statistical power of this finding should be interpreted with caution. Regarding sexual status, non-pregnant jennies had higher serum tT4 concentrations than pregnant ones. In mares, gestation affected tT3, with higher concentrations during the first trimester of gestation [51]. Although the effects of libido and breeding season on TH concentrations have been described in jacks [47], these effects could not be evaluated in our study because only intact jacks were included. Regarding age, although younger donkeys had higher serum TH concentrations, statistically significant differences were not observed in this study, contrary to a previous one using RIA [10]. Multiple discrepancies among studies have been observed in horses, with some authors reporting no effect of age on TH [40,52], while other studies demonstrated differences [53], all using RIA. Similar to the gender, since small subgroups were compared, these findings could be of limited statistical power. The effect of preanalytical factors should also be taken into consideration. In our study, the time from collection to centrifugation, separation, and storage was less than 6 h, and only one aliquot was used for each determination (biochemistry or hormone measurements). Nonetheless, TH concentrations have been reported to be stable during long-time frozen storage and after several thaw–freeze cycles [54].

The present study has some limitations and weaknesses, mainly related to the composition of the groups. While differences between our donkeys and horses could have been influenced by dissimilarities in age and gender between both populations, the Quade ANCOVA analysis discarded this effect as a possible bias in our results, although it does not fully eliminate this potential confounding effect. Additionally, since some of our age and sex cohorts were small, differences between these groups should be taken with caution. As most animals in our study were of the Andalusian breed, the findings in this report should only apply to the population studied. Reference intervals (RIs) for TH in donkeys were not established in this study due to the low number of animals included, as the American Society of Veterinary Clinical Pathology recommends the inclusion of at least 150 healthy individuals to establish accurate RIs [55].

## 5. Conclusions

The Immulite 2000xpi yielded lower CVs and fewer measurements under the LOD for tT4 and fT4 determinations compared to tT3 and fT3. Further in-depth validation of this analyzer is required in equids to confirm or discard the analytical accuracy and clinical applicability of this analyzer, particularly for TSH using the canine-specific assay. Since TH concentrations are different between donkeys and horses, species-specific and technique-specific ranges should be used by clinicians evaluating thyroid gland disturbances in equids.

## Figures and Tables

**Table 1:** Assay performance for each kit according to the manufacturer and previous reports [16,17,18].

	Analytical Sensitivity	Measuring Range	CV	Kit
tT4	0.12 μg/dL	0.5–15 μg/dL	<12%	Canine total T4 kit, 104 KT4
fT4	0.04 ng/dL	0.3–6 ng/dL	<11.9%	Veterinary free T4 kit, VF4
tT3	19 ng/dL	40–600 ng/dL	<15%	Total T3 kit, T3
fT3	1 pg/mL	1–40 pg/mL	<10%	Free T3 kit, FT3
TSH	0.01 ng/mL	0.03–12 ng/mL	<10%	Canine TSH kit

CV, Intra- and inter-assay coefficient variation; TSH, thyroid-stimulating hormone; fT3: free triiodothyronine; fT4: free thyroxine; tT3: total triiodothyronine; tT4: total thyroxine.

**Table 2:** Thyroid hormone concentrations in healthy donkeys and horses analyzed by chemiluminescence immunoassay technique (Immulite 2000xpi).

Thyroid Hormone	Donkeys Median (IQR) 95% Confidence Interval (Range)	Horses Median (IQR) 95% Confidence Interval (Range)	*p* Values Age-Adjusted ANCOVA	*p* Values Gender-Adjusted ANCOVA
tT4 (µg/dL)	3.2 (1.6) ** 2.9–4.3 (0.5–9.3)	1.5 (1.1) 1.4–1.8 (0.5–3.0)	<0.001	0.003
fT4 (ng/dL)	1.3 (0.4) ** 1.2–1.5 (0.7–2.5)	0.8 (0.7) 0.7–1.0 (0.3–1.7)	0.001	0.030
tT3 (ng/dL)	59 (27) * 57–77 (40–147)	46 (14) 40–56 (40–62)	0.085	0.387
fT3 (pg/mL)	1.5 (0.9) 1.5–2.2 (1.1–4.4)	1.5 (0.7) 1.3–2.1 (1.0–3.2)	0.924	0.854

Data are expressed as median (IQR, interquartile range), lower and upper 95% confidence intervals, and range (minimum–maximum measured concentrations). fT3: free triiodothyronine; fT4: free thyroxine; tT3: total triiodothyronine; tT4: total thyroxine. * *p* < 0.05 vs. horses; ** *p* < 0.01 vs. horses.

**Table 3:** Thyroid hormone concentrations in healthy donkeys grouped by age.

Thyroid Hormone	Group 1: <5 Years Old Median (IQR) 95% Confidence Interval (Range)	Group 2: 5–10 Years Old Median (IQR) 95% Confidence Interval (Range)	Group 3: >10 Years Old Median (IQR) 95% Confidence Interval (Range)
tT4 (µg/dL)	3.6 (2.71) 3.1–5.3 (1.4–9.3)	2.8 (1.4) 2.2–4.2 (0.5–7.7)	3.0 (0.8) 2.6–3.59 (2.2–3.8)
fT4 (ng/dL)	1.3 (0.7) 1.2–1.7 (0.7–2.5)	1.2 (0.4) 1.0–1.3 (0.7–1.5)	1.0 (0.6) 0.8–1.8 (0.8–2.4)
tT3 (ng/dL)	55.4 (51.6) 52.4–92.2 (40.2–147.0)	60.0 (24.0) 49.5–73.3 (40.9–101.0)	64.2 (21.9) 48.1–85.2 (56.1–82.0)
fT3 (pg/mL)	2.1 (1.1) 1.5–2.5 (1.1–3.4)	1.5 (0.9) 1.1–2.7 (1.2–4.4)	1.4 (1.0) 0.7–2.4 (1.1–2.3)

Data are expressed as median (IQR, interquartile range), lower and upper 95% confidence intervals, and range (minimum–maximum measured concentrations). fT3: free triiodothyronine; fT4: free thyroxine; tT3: total triiodothyronine; tT4: total thyroxine.

**Table 4:** Thyroid hormone concentrations in donkeys according to gender.

Thyroid Hormone	Jacks Median (IQR) 95% Confidence Interval (Range)	Jennies Median (IQR) 95% Confidence Interval (Range)	*p* Values
tT4 (µg/dL)	3.5 (3.9) 2.3–6.0 (2.1–3.9)	3.1 (1.6) 2.9–4.2 (0.5–9.3)	0.19
fT4 (ng/dL)	1.4 (0.6) 1.0–1.9 (0.7–2.5)	1.2 (0.4) 1.1–1.5 (0.7–2.4)	0.18
tT3 (ng/dL)	53.0 (33.3) 41.2–80.2 (41.8–100.0)	62.90 (27.1) 57.0–81.9 (40.2–147.0)	0.22
fT3 (pg/mL)	1.4 (0.9) 1.1–2.0 (1.1–2.2)	1.7 (1.1) 1.5–2.4 (1.0–4.4)	0.14

Data are expressed as median (IQR, interquartile range), lower and upper 95% confidence intervals, and range (minimum–maximum measured concentrations). fT3: free triiodothyronine; fT4: free thyroxine; tT3: total triiodothyronine; tT4: total thyroxine.

## Data Availability

The original contributions presented in this study are included in the article/Supplementary Material. Further inquiries can be directed to the corresponding authors.

## Supplementary Materials

The following supporting information can be downloaded at https://www.mdpi.com/article/10.3390/vetsci13070690/s1, Table S1: Hematology results in donkeys (n = 40) and horses (n = 41) included in this study; Table S2: Biochemistry results in donkeys (n = 40) and horses (n = 41) included in this study; Table S3: Thyroid hormone concentrations in healthy donkeys and horses grouped according to sexual status.
