# Supplementary material for: Assessment of Thyroid Hormones Using the Immulite 2000xpi Analyzer in Healthy Donkeys
Source: Vet Sci. 2026 Jul 15;13(7):690. doi: 10.3390/vetsci13070690 (PMC13419001; doi:10.3390/vetsci13070690)
Supplement: Supplementary file 1 [file vetsci-13-00690-s001.zip › Table S1 -J2.pdf]

Table S1. Hematology results in donkeys (n=40) and horses (n=41) included in this study.

Parameter	RBC ($10^6/\mu L$)	Hb (g/dL)	HTC (%)	WBC ($10^3/\mu L$)	NEU ($10^3/\mu L$)	NEU (%)	LYM ($10^3/\mu L$)	LYM (%)	MONO ($10^3/\mu L$)	MONO (%)	EOS ($10^3/\mu L$)	EOS (%)	BASO ($10^3/\mu L$)	BASO (%)	PLT ($10^3/uL$)
**Donkeys**	6.7 (1)<br>6.5-7.5	13.0 (2)<br>12.6-14.1	36.1 (4)<br>35.4-39.3	10.2 (4)<br>9.5-11.9	5.3 (2)<br>4.7-6.1	49.3 (12)<br>47.0-53.5	3.9 (2)<br>3.6-4.9	40.4 (10)<br>36.2-42.9	0.6 (0)<br>0.5-0.7	5.6 (1)<br>5.3-6.3	0.3 (1)<br>0.3-0.5	3.5 (5)<br>2.7-4.8	0.05 (0)<br>0.04-0.08	0.5 (0)<br>0.4-0.8	239 (89)<br>198.4-253.9
Reference range*	5.2 – 7.4	9.4 – 14.9	27 – 42	6 – 15	2.2 – 6.5	35 – 68	2.1 – 7.8	18 – 51	0.3 – 1.5	3 – 14	0 – 0.9	2 – 9	0 – 0.1	0 – 0.5	93 - 350
**Horses**	8.1 (1.6)<br>7.6-8.3	12.4 (2.2)<br>12.5-13.5	35.4 (6.4)<br>34.9-37.9	7.5 (2.4)<br>6.9-8.2	4.5 (1.3)<br>4.1-4.8	59.9 (8.1)<br>57.4-61.5	2.5 (1.0)<br>2.2-2.8	31.7 (5.9)<br>30.1-34.2	0.3 (0.1)<br>0.3-0.4	4.7 (1.4)<br>4.6-5.2	0.2 (0.2)<br>0.2-0.3	2.7 (2.1)<br>2.4-3.9	0.03 (0.02)<br>0.02-0.03	0.4 (0.3)<br>0.3-0.4	137 (35)<br>122.4-142.3
Reference range*	6 - 11	10.5 - 17	30 - 43	6 - 12	2.5 - 7.5	30 - 70	1.5 - 5	15 - 50	0.2 - 0.5	0 - 7	0 - 0.4	0 - 3	0 - 0.1	0 - 1	90 - 300

Data are expressed as median (IQR, interquartile range) and below in the second line 95% confidence interval. BASO, basophils; EOS, eosinophils; Hb, hemoglobin; HTC, hematocrit; LYM, lymphocytes; MONO, monocytes; NEU, neutrophils; PLT, platelets; RBC, red blood cells; WBC, white blood cells. * Reference ranges internally established in our laboratory.
