# Supplementary material for: Assessment of Thyroid Hormones Using the Immulite 2000xpi Analyzer in Healthy Donkeys
Source: Vet Sci. 2026 Jul 15;13(7):690. doi: 10.3390/vetsci13070690 (PMC13419001; doi:10.3390/vetsci13070690)
Supplement: Supplementary file 1 [file vetsci-13-00690-s001.zip › Table S2 -J2.pdf]

Table S2. Biochemistry results in donkeys (n=40) and horses (n=41) included in this study.

Parameter	GLDH (UI/L)	LDH (UI/L)	CK (UI/L)	AST (UI/L)	GGT (UI/L)	ALP (UI/L)	TB (mg/dL)	GLO (g/dL)	Albumin (g/dL)	TP (g/dL)	CREA (mg/dL)	URE (mg/dL)	TGL (mg/dL)	GLU (mg/dL)
**Donkeys**	5.2 (1.8)<br>4.7-6.0	145.4 (168.4)<br>87-207	83.0 (38.8)<br>76.2-94.48	220.7 (59.6)<br>208.9-241.6	20.8 (8.9)<br>19.5-25.1	393.3 (121.2)<br>364.1-466.5	0.2 (0.1)<br>0.2-0.3	3.0 (0.7)<br>2.8-3.2	3.4 (0.4)<br>3.2-3.5	6.3 (0.5)<br>6.1-6.6	1.2 (0.4)<br>1.1-1-3	20.1 (18.9)<br>18.1-25.2	52.1 (49.2)<br>49.3-83.7	80.7 (9.0)<br>79.3-84.8
Reference range*	< 10	0 - 600	50 - 300	200 - 400	10 - 75	100 - 500	0,1 - 1	2,5 - 4,5	2,2 - 4	5,5 - 7,5	0,6 - 1,7	11 - 44	15 - 150	70 - 90
**Horses**	4.7 (1.4)<br>4.2-5.4	3.1 (3.3)<br>11.3-125.5	123.5 (40.6)<br>126.1-154.2	252.6 (66.9)<br>251.9-278.3	16.4 (6.2)<br>15.7-20.5	343.9 (150.5)<br>329.7-406.8	1.5 (0.4)<br>1.4-1.6	2.3 (0.6)<br>2.1-2.4	3.7 (0.3)<br>3.6-3.7	5.9 (0.5)<br>5.8-6.1	1.6 (0.3)<br>1.5-1.6	23.2 (11.8)<br>20.9-27.4	35.0 (18.2)<br>31.9-39.7	84.1 (9.2)<br>83.7-88.3
Reference range*	< 12	0 - 800	50 - 350	50 – 350	5 - 35	50 - 550	0.3 - 3	2 – 4	2.5 - 4	5.5 – 7.5	0.8 – 2	15 – 45	20 - 50	80 – 110

Data are expressed as median (IQR, interquartile range) and below in the second line 95% confidence interval. ALB, albumin; ALP, alkaline phosphatase; AST, aspartate aminotransferase; CK, creatine kinase; CREA, creatinine; GGT, gamma-glutamyl transferase; GLDH, glutamate dehydrogenase; GLO, globulin; GLU, glucose; LDH, lactate dehydrogenase; TB, total bilirubin; TGL, triglycerides; TP, total proteins; URE, urea. *Reference ranges established internally in our laboratory.
