# Supplementary material for: Assessment of Thyroid Hormones Using the Immulite 2000xpi Analyzer in Healthy Donkeys
Source: Vet Sci. 2026 Jul 15;13(7):690. doi: 10.3390/vetsci13070690 (PMC13419001; doi:10.3390/vetsci13070690)
Supplement: Supplementary file 1 [file vetsci-13-00690-s001.zip › Table S3 -J2.pdf]

Table S3. Thyroid hormone concentrations in healthy donkeys and horses grouped according to sexual status.

Thyroid hormone	Jennies			Horses		
	**Non-pregnant**	**Pregnant**	*p* values	**Stallions**	**Geldings**	*p* values
tT4 (μg/dL)	4.31 (2.46)*	2.90 (0.87)	0.012	1.34 (1.55)	1.37 (0.95)	0.841
fT4 (ng/dL)	1.40 (0.31)	1.12 (0.43)	0.299	0.73 (0.49)	0.55 (0.27)	0.406
tT3 (ng/dL)	65.80 (50.70)	48.40 (46.97)	0.075	20.0 (0)	20.0 (26.0)	0.211
fT3 (pg/mL)	1.37 (1.63)	0.77 (0.96)	0.435	0.50 (0.49)	1.30 (1.28)	0.099

Data are expressed as median (IQR, interquartile range). fT3: free triiodothyronine; fT4: free thyroxine; tT3: total triiodothyronine; tT4: total thyroxine.
[a] $p < 0.05$ vs pregnant.
